# Towards an In Vitro Retinal Model to Study and Develop New Therapies for Age-Related Macular Degeneration

**DOI:** 10.3390/bioengineering8020018

**Published:** 2021-01-22

**Authors:** Beatrice Belgio, Federica Boschetti, Sara Mantero

**Affiliations:** Department of Chemistry, Materials and Chemical Engineering “Giulio Natta”, Politecnico di Milano, 20133 Milan, Italy; federica.boschetti@polimi.it (F.B.); sara.mantero@polimi.it (S.M.)

**Keywords:** retina, 3R, in vitro model, ophthalmology, age-related macular degeneration, biomechanics, electrospinning

## Abstract

Age-related macular degeneration (AMD) is the leading cause of vision loss in the elderly worldwide. So far, the etiology and the progression of AMD are not well known. Animal models have been developed to study the mechanisms involved in AMD; however, according to the “Three Rs” principle, alternative methods have been investigated. Here we present a strategy to develop a “Three Rs” compliant retinal three-dimensional (3D) in vitro model, including a Bruch’s membrane model and retina pigment epithelium (RPE) layer. First, tensile testing was performed on porcine retina to set a reference for the in vitro model. The results of tensile testing showed a short linear region followed by a plastic region with peaks. Then, Bruch’s membrane (BrM) was fabricated via electrospinning by using *Bombyx mori* silk fibroin (BMSF) and polycaprolactone (PCL). The BrM properties and ARPE-19 cell responses to BrM substrates were investigated. The BrM model displayed a thickness of 44 µm, with a high porosity and an average fiber diameter of 1217 ± 101 nm. ARPE-19 cells adhered and spread on the BMSF/PCL electrospun membranes. In conclusion, we are developing a novel 3D in vitro retinal model towards the replacement of animal models in AMD studies.

## 1. Introduction

Age-related macular degeneration (AMD) is the commonest cause of blindness in high-income countries. It affects globally between 30 and 50 million people, and, as populations age, the number of patients is predicted to rise [1]. AMD is a neurodegenerative disease triggered by age-related changes in the retinal pigment epithelium (RPE) and in its basement Bruch’s membrane (BrM) at the macular region. These changes first manifest with the accumulation of cellular debris called drusen between the RPE and its BrM, and the thickening of the BrM along with abnormalities of the RPE [2]. Then, AMD can progress to a “dry”, non-vascular form that leads to geographic atrophy of the RPE and, subsequently, the overlying of the photoreceptors, or to a “wet”, neovascular form, which occurs when aberrant choroidal blood vessels penetrate the BrM and grow through the RPE, resulting in vascular leakage, hemorrhage, and fibrosis [3]. Currently, treatments are available and evolving for the “wet” form of AMD, most notably intravitreal injections of anti-vascular endothelial growth factor (VEGF) drugs, whereas there is as yet no effective cure for the “dry” form of AMD. Moreover, even though anti-VEGF-based treatments have been demonstrated to halve the rate of blindness slowing down the progression of the wet AMD, they are expensive, represent a significant burden to patients and caregivers, and do not address the underlying disease processes, nor restore tissue functionality [4]. This relates to the fact that AMD is a complex, multifactorial, and heterogenous disease, with both environmental and genetic risk associations, and we still do not fully understand the pathogenic mechanisms involved in AMD [5]. As such, it is fundamental to study and acquire greater insight into the underlying pathways involved in this disease to foresee novel treatments on the horizon.

So far, animal models have been used to study AMD progression and evaluate the safety and efficacy of new therapies because they replicate several of the important pathological features seen in AMD [6]. In addition, retinal degeneration may be induced in animals under controlled conditions and in relatively short amounts of time [7]. Experimental in vivo models of AMD have been developed in many species, including drosophila, mice, rats, guinea pigs, rabbits, pigs, and non-human primates [6,7]. However, high-scale in vivo methods are relatively complex to establish, thus not allowing high-throughput drug screenings in a reasonable time at a reasonable cost. Even more importantly, the ethical concern for animal welfare and the widespread adoption of the “Three Rs” guideline, defined as refine, reduce, and replace, have ushered in the use of alternative approaches and protocols [8]. In fact, in the EU, the number of animals used for research and testing is significantly decreasing over the years. The total number of animals from the data collected in 2011 was just under 11.5 M, whilst the number of animals reported from 2015 to 2017 was below 10 M, with a decrease also continuing between 2015 and 2017 [9].

An interesting and attractive alternative to in vivo studies is three-dimensional (3D) in vitro tissue models. In these 3D in vitro models, human cells are employed to create an organotypic tissue that recapitulates aspects of the in vivo microenvironment. Nowadays, there are no suitable in vitro models for the retina to study the effects of retinal diseases, such as AMD, or to test new treatments [10]. Many attempts to reconstruct retinal tissue in vitro have been made, starting from the RPE cellular layer [11]. It has been demonstrated that RPE cellular viability and functionality improve when cultured on specific support that mimics native BrM [11]. In addition, the BrM is also compromised in AMD. As such, the development of a BrM model is necessary to obtain a more accurate in vitro model of AMD. In most studies, two-dimensional (2D) membranes with a closed dense structure have been used as BrM models [12,13,14]. Nevertheless, such a structure contrasts with the open fibrillar structure of a native BrM and can prevent nutrient diffusion. Furthermore, cells accomplish their function in a 3D environment that resembles their natural habitat [15]. Recently, electrospinning has been proposed as a promising technique to fabricate a BrM model as it allows us to generate 3D nanofibrous network topographies that are highly permeable for solutes, thus facilitating cell adhesion and proliferation [16]. In particular, electrospun polycaprolactone (PCL) nanofibers have been proven to both have good mechanical properties and support the growth and proliferation of retinal cells [17,18]. However, PCL hydrophobicity, and thus the lack of recognition sites for cell attachment, limit its use as a BrM material. Previous studies showed that cell attachment may be improved by blending PCL with silk fibroin (SF) [18,19].

The goal of our research project is to develop a suitable “Three Rs”-compliant three-dimensional (3D) in vitro model to partially replace in vivo studies on the mechanisms involved in AMD. To better replicate the in vivo conditions, our model will include a BrM model.

Retinal mechanical properties are yet to be determined as they have been scarcely investigated due to retinal extreme structural fragility [20]. As such, in this study, we first investigated the biomechanical properties of porcine retina to build an in vitro model that resembles the native retinal tissue. To this end, in line with the principle of the “Three Rs”, we collected pig eyes from a local abattoir applying the reuse of animals, thus reducing the total number of animals used for research purposes. Then, we started developing the in vitro model from the fabrication of the BrM model for RPE cell culture. We selected a blend of PCL and SF to fabricate a scaffold that mimics the topological and mechanical cues of human BrM, and we characterized our BrM scaffold in terms of morphology, mechanical properties, permeability, and biocompatibility.

## 2. Materials and Methods

### 2.1. Porcine Sample Collection and Preparation

**Figure 1:**
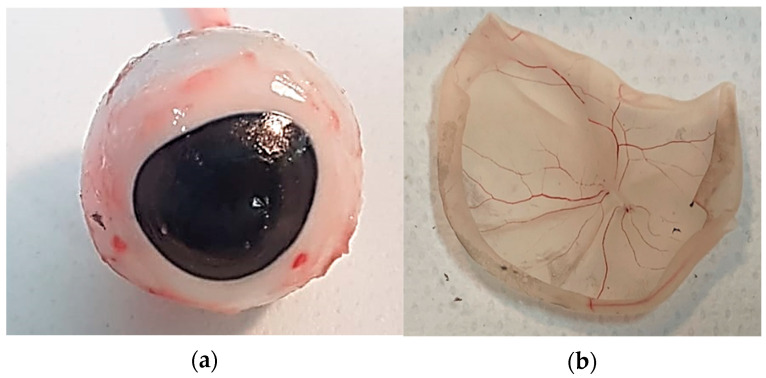
(**a**) Clean eyeball after adipose tissue and conjunctiva removal; (**b**) detached retina preserved in phosphate buffer saline solution (PBS).

Porcine eyes were collected from a local slaughterhouse (Fumagalli Industria Alimentare, Tavernerio (CO), Italy), within 1 h postmortem. The average age of the pigs was 9 months. After cleaning the eyeballs of adipose tissue and conjunctiva (Figure 1a), we made a circular cut on the sclera at the cornea level with a scalpel. Then, we carefully removed in sequence cornea, iris, crystalline lens, and vitreous to isolate the retina. The isolated retina was cautiously detached from the posterior wall of the posterior chamber (Figure 1b), and preserved in phosphate buffer saline solution (SIGMA, PBS, P4417, St. Loius, MO, USA) at 4 °C until testing to avoid tissue dehydration and degradation. Retinal samples for mechanical measurements were obtained by dissecting one retinal strip with equatorial orientation per eye from the center of the retina using a scalpel.

### 2.2. Biomechanical Measurements of Porcine Retina

**Figure 2:**
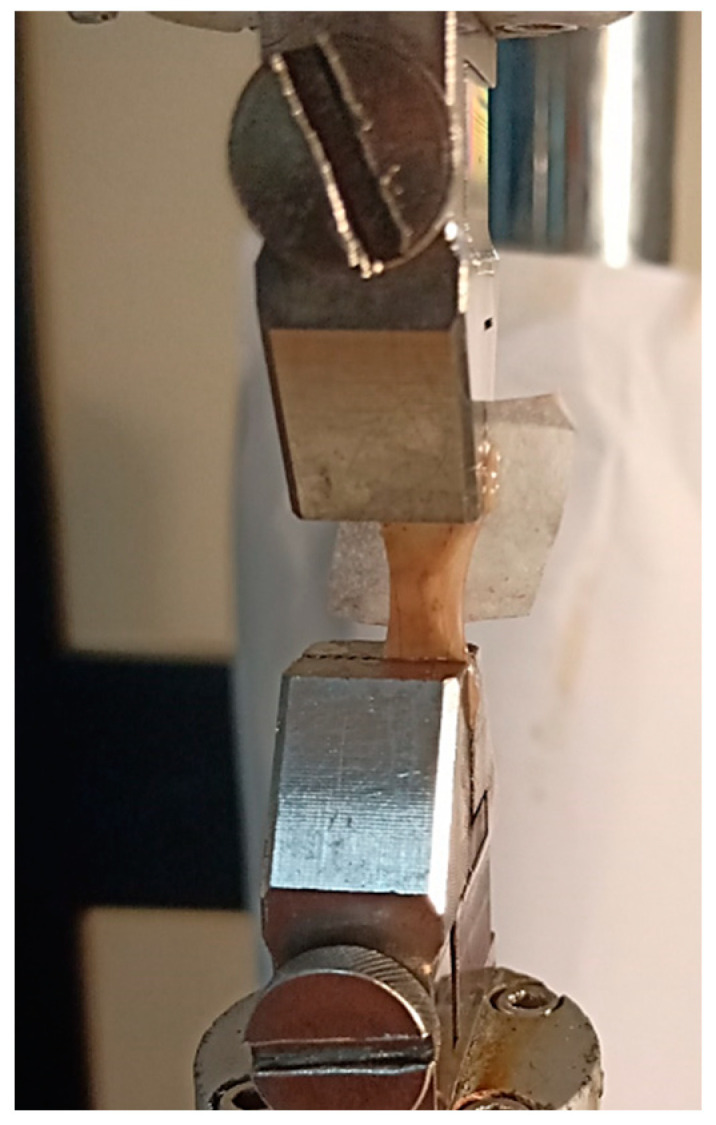
Retinal samples clamped between the grips of the testing machine.

For the measurements, retinal samples were clamped between the jaws of an electromechanical testing machine (Bose EnduraTEC ELF 3200 Uniaxial Testing System, Eden Prairie, MN, USA) equipped with a 22 N load cell (Figure 2). Grips were made of knurled stainless steel to prevent the slippage of retinal specimens. Samples were pulled until failure at a velocity of 0.1 mm/s at room temperature. Sixteen samples (*n* = 16) were tested. The sample size was arbitrarily chosen to reduce inter-sample variability.

For the analysis, we used the force (N)—elongation (mm) data to compute the stress (MPa) defined as the ratio of force applied F (N) to the original cross section A (mm^2^), as indicated in Equation (1), and the strain (mm/mm), defined as the change of length ΔL (mm) divided by the original length L_0_ (mm), as in Equation (2).

Stress = F/A(1)

Strain = ΔL/L_0_ (2)

The gap between the clamps was considered as the original length. The initial width of the samples was evaluated through the ImageJ software (National Institutes of Health, Bethesda, MD, USA). The thickness of the retina, measured from three porcine eyes (*n* = 3), was determined using optical coherence tomography (OCT) (DRI OCT Triton Plus, Topcon, Tokyo, Japan). The original cross section area was calculated as the thickness multiplied by the initial width. The elastic modulus (E) was extrapolated from the stress–strain curves as the linear portion of the curve before the change of the slope. The yield stress (Y) was considered as the point of change of slope in the first linear elastic region of the stress–strain curve. The failure stress was set to the maximum stress value.

### 2.3. Electrospinning Process

Silk fibroin was extracted from *Bombyx mori* silk cocoon (Crea, Roma, Italy) according to a patented protocol (Leonardino s.r.l.). The polymer solutions with a concentration of 15% (wt/v) were derived by dissolution of *Bombyx mori* silk fibroin (BMSF) and Polycaprolactone (PCL, 440744-250G, Sigma, St. Louis, MO, USA) with a weight ratio of 5:95 (BMSF/PCL) in 98% formic acid (Sigma, St. Louis, MO, USA). The BMSF/PCL nanofibrous scaffolds were fabricated using the EF300 electrospinning system (SKE Research Equipment, Leonardino s.r.l.). A voltage of 18 kV was applied to the syringe needle 15 cm distant from the surface of the collector, which was grounded. The polymer solution was delivered at a feeding rate of 1.3 mL/h. Electrospinning was performed at a constant temperature (33.5 °C) and a relative humidity of 22%.

### 2.4. BMSF/PCL Scaffold Characterization

Scaffold physio-chemical and mechanical properties, including physical morphology, stress–strain relation, elasticity, and permeability, were examined.

To investigate the surface topography, scaffolds were sputter-coated with gold and observed with a scanning electron microscope (SEM, Stereoscan 360, Cambridge Instruments, Cambridge, UK) at 10 kV. The fiber diameter and the packing density were assessed through image elaboration with ImageJ software (National Institute of Health, Bethesda, MD, USA). Twenty random fiber diameters were measured to calculate the average fiber diameter. The packing density, presented as a percentage, was computed by counting the number of the fibers across each image, multiplying this by the average fiber diameter and then dividing by the width of the image.

For the mechanical measurements, samples were prepared by cutting the scaffolds into ten 5 × 32 mm samples (*n* = 10). Sample size was arbitrarily chosen. A material testing machine (Synergie 200, MTS Systems, Eden Prairie, MN, USA) equipped with a 100 N loading cell was used to pull five samples until failure at a displacement rate of 0.1 mm/s. Samples were first preconditioned four times and then pulled to failure. The same protocol was applied to five hydrated samples, i.e., samples submerged in saline solution for 1 h. Elastic modulus (E) was extracted from the stress–strain plots as the slope of the initial linear region of the stress–strain curve.

**Figure 3:**
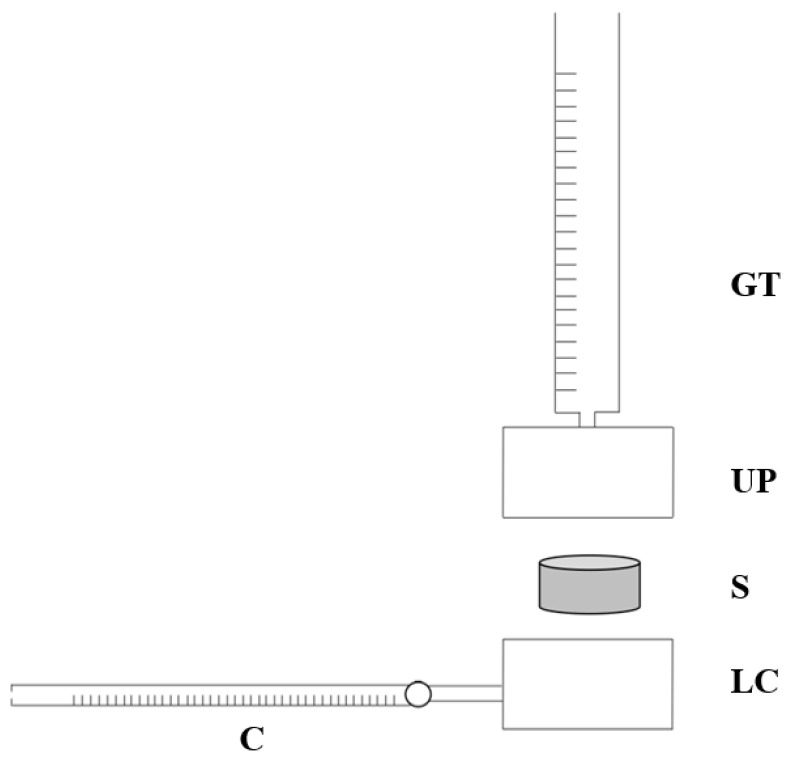
Scheme of the custom-made permeability set-up. Shown in the figure are the graduated tube (**TG**), upper (**UC**) and lower (**LC**) chambers, capillary (**C**), and scaffold sample (**S**).

A custom-made apparatus was employed to evaluate the permeability of the BMSF/PCL scaffolds. The system consists of two coaxial stainless-steel cylinders (upper and lower chambers), a polyethylene filter, an O-Ring, a graduated tube, and a capillary flow-meter with a resolution of 10^−3^ mL (Figure 3). O-Ring guarantees the seal between the two cylinders. Three samples (*n* = 3) of 10 mm diameter were obtained with a 10 mm biopsy punch. Sample size was arbitrarily chosen.

Briefly, after inserting the sample properly into a cavity of 10 mm between the two cylinders, we applied a constant hydrostatic pressure to the sample and measured the fluid volume through the sample over time. The hydrostatic pressure was applied as hydraulic head by a graduated tube connected to the upper chamber. The fluid flows into the capillary flow-meter after passing through the constrained sample according to its permeability. The permeating fluid was a physiologic solution. The following four different values of pressure drop were applied to each sample: 10 cm H_2_O, 20 cm H_2_O, 30 cm H_2_O, 40 cm H_2_O (corresponding to 981 Pa, 1962 Pa, 2943 Pa, 3924 Pa). Samples were allowed to recover for 10 min between tests. For each pressure value, the time Δt taken by the fluid to cover ΔV = 3 µL in the capillary flowmeter was recorded. Permeability was calculated using Darcy’s law, as in Equation (3):

k = (ΔV × µ × h)/(Δt × A × ΔP)(3)

where k is the permeability, ΔV/Δt is the fluid volume over time, µ is the fluid viscosity, h is the sample thickness, A is the flow area, and ΔP is the applied hydrostatic pressure. Darcy’s permeability was then normalized by the sample thickness t and the fluid viscosity µ to obtain hydraulic conductivity L_p_, as in the following Equation (4):

L_p_ = k/(µ × t) (4)

### 2.5. ARPE-19 Cell Culture and Seeding

ARPE-19 cells, a human retinal pigment epithelium cell line, were obtained from American Type Culture Collection, Manassas, VA, USA (Catalog # CRL-2302™, ATCC). ARPE-19 was routinely cultured in complete medium composed of DMEM/F12 (Catalog # 30-2006™, ATCC^®^) with 10% fetal bovine serum (Catalog # 20-2020™, ATCC^®^) and 1% penicillin/streptomycin (Catalog # 15140122, Gibco™, Gaithersburg, MD, USA) at 37 °C and 5% CO_2_. Scaffolds of 20mm × 20mm BMSF/PCL were soaked in 70% ethanol for 24 h followed by washing three times with sterile phosphate saline buffer (PBS, Catalog # 20012027, Gibco™, Gaithersburg, MD, USA). Scaffolds were transferred to 60 mm × 15 mm Petri dishes and incubated in complete medium at 37 °C and 5% CO_2_ overnight for preconditioning. ARPE-19 cells were seeded at a density of 4 × 10^5^ cells/mL. For cell adhesion evaluation, cells were grown for five days in complete medium at 37 °C and 5% CO_2_ and complete medium was changed every two days.

### 2.6. Adhesion and Morphology of ARPE-19 Cells on BMSF/PCL Electrospun Scaffolds

The adhesion and the morphology of ARPE-19 cells on the BMSF/PCL scaffolds were assessed using SEM (Stereoscan 360, Cambridge Instruments, Cambridge, UK). On day 5, after culture medium removal, the cell-seeded scaffolds were rinsed with PBS and fixed in 3% glutaraldehyde for 2 h at room temperature. The samples were then dehydrated through a concentration gradient of 20%, 50%, 70%, 90%, and 100% ethanol. The same were sputter-coated with gold and observed with SEM at 10 kV.

### 2.7. Statistical Analyses

Results are shown as mean ± standard deviation (SD). The unpaired t-test was performed for the statistical analyses. Differences were considered statistically significant if the *p*-value was less than 0.05.

## 3. Results

### 3.1. Stress–Strain Measurements of Porcine Retina

**Figure 4:**
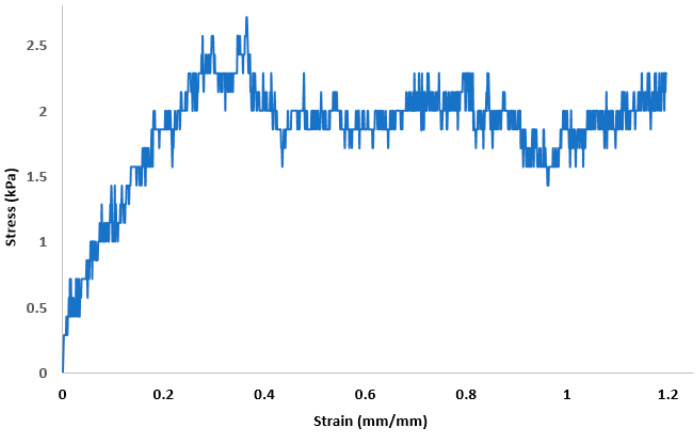
Tensile stress versus strain curve of retinal specimens at room temperature.

The average sample thickness was 0.35 ± 0.04 mm (*n* = 3). For the stress–strain measurements of retinal tissue, sixteen samples (*n* = 16) were tested, and their resulting curves analyzed to extrapolate the biomechanical parameters considered, namely E, Y, and failure stress. Figure 4 displays a typical tensile stress–strain curve of retinal samples. The curve presents a narrow initial linear portion followed by a wide region of plastic behavior before rupture (Figure 4). The plastic region of the curve is characterized by several peaks probably due to micro-ruptures in the tissue. From the analysis of the curves, the average E at room temperature was 13.4 ± 0.0067 kPa. The Y and the failure stress were on average 1.33 ± 0.75 kPa and 2.21 ± 0.8 kPa, respectively.

### 3.2. BMSF/PCL Scaffold Characterization

**Figure 5:**
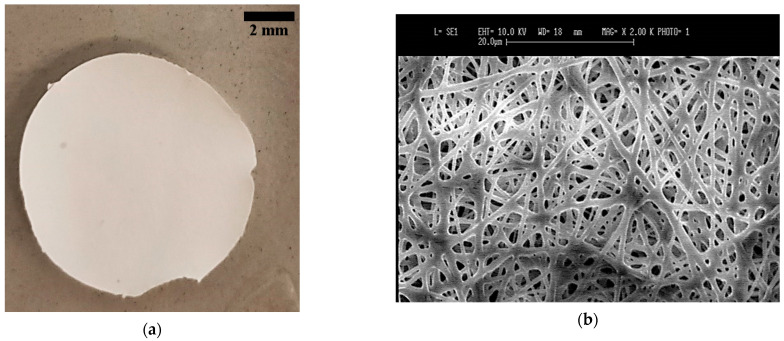
(**a**) Appearance of electrospun BMSF/PCL scaffolds after fabrication before the immersion in ethanol or PBS. Scale bar = 2 mm; (**b**) SEM micrographs of electrospun BMSF/PCL scaffolds (top view). Scale bar = 20 µm.

The scaffolds for the BrM model composed of BMSF and PCL were successfully fabricated by electrospinning and appeared to be uniform (Figure 5a). SEM images showed that the scaffolds were constructed of randomly oriented fibers and thoroughly interconnected pores (Figure 5b). The electrospun scaffolds displayed a thickness of 44 μm, an average fiber diameter of 1217 ± 101 nm, and a fiber packing density of 63.76 ± 1.2%. The fibers in the fabricated scaffolds showed structural similarity to those of the inner collagenous layer of human BrM, according to the findings of Warnke et al. [16]. The inner collagenous layer of human BrM presents a structural network of randomly organized fibers with a thickness of 2–4 µm, 60 nm fiber diameter, and a 48% packing density [16].

**Figure 6:**
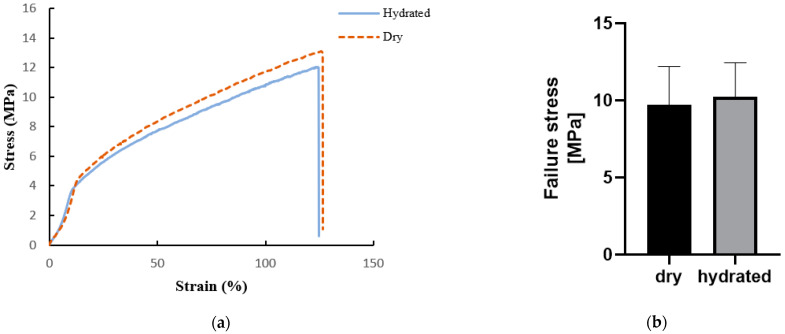

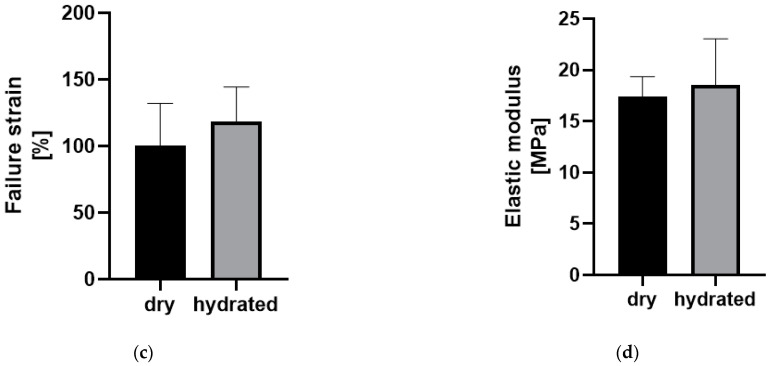
(**a**) Stress versus strain curve of BMSF/PCL hydrated (black continuous line) and dry (dotted orange line) scaffolds under tensile test; (**b**) failure stress of dry (black column) and hydrated (grey column) scaffolds; (**c**) failure strain of dry (black column) and hydrated (grey column) scaffolds; (**d**) elastic modulus (E) of dry (black column) and hydrated (grey column) scaffolds.

Regarding the mechanical properties, a typical tensile stress–strain curve was obtained for both the dry (*n* = 5) and hydrated (*n* = 5) scaffolds (Figure 6a). The curves displayed an initial linear region followed by plastic deformation before break. From the analysis of the curves, the failure stress, the failure strain, and the elastic modulus E were extrapolated. The failure stress of the dry scaffold was 9.72 ± 2.47 MPa, with a failure strain of 100.4 ± 31.6% and an elastic modulus of 17.37 ± 1.99 MPa. The hydrated scaffold had slightly higher values of failure stress, failure strain, and E, of 10.234 ± 2.21 MPa, 118.6 ± 25.9%, and 18.57 ± 4.47 MPa, respectively. However, no significant difference was found between the mechanical parameters of dry and hydrated scaffolds (Figure 6b–d).

**Figure 7:**
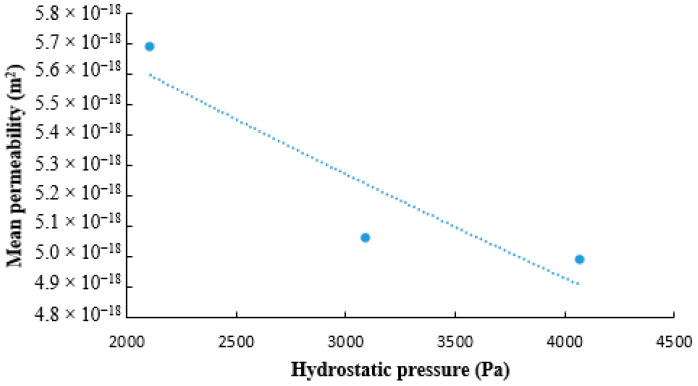
Curve of average permeability of BMSF/PCL scaffold versus hydrostatic pressure.

Scaffold permeability was assessed for three samples (*n* = 3) to evaluate how a fluid flows through the material. We calculated the average permeability coefficient through Darcy’s law for each applied hydrostatic pressure. As shown in Figure 7, the average permeability coefficient decreased with the increase in the hydrostatic pressure (Figure 7). The hydraulic conductivity was computed as in equation (4), and was shown to be around 20 × 10^−10^ m/(Pa·s). These results are in agreement with those of Moore et al., who found that the hydraulic conductivity of Bruch’s membrane ranges from 20 to 100 × 10^−10^ m/(Pa·s) [21].

### 3.3. Adhesion and Morphology of ARPE-19 Cells on BMSF/PCL Electrospun Scaffolds

**Figure 8:**
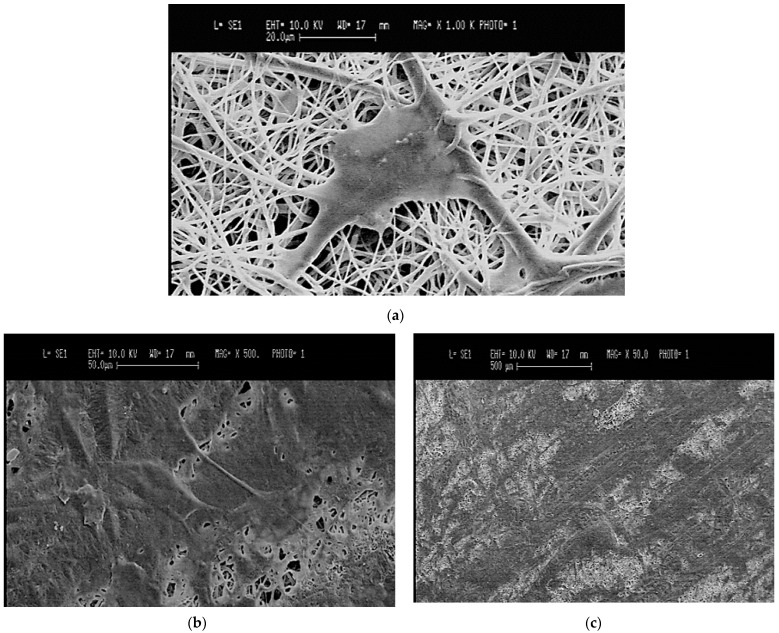
(**a**) SEM image of an ARPE-19 cell on BMSF/PCL scaffold 5 days after seeding (scale bar = 20 µm); (**b**) SEM image of ARPE-19 cells on BMSF/PCL scaffold 5 days after seeding (scale bar = 50 µm); (**c**) SEM image of an ARPE-19 cell on BMSF/PCL scaffold 5 days after seeding (scale bar = 500 µm).

To visualize the adhesion and the morphology of the ARPE-19 cells seeded on electrospun scaffolds, SEM images were taken after 5 days in culture. Cells adhered well to the sample surfaces, as demonstrated by the flattened cellular morphology with filopodia anchored on the sample nanostructures (Figure 8a). Moreover, at 5 days post-seeding, cells were observed spreading on sample surfaces, resulting in a good cell colonization of the scaffold (Figure 8b,c).

## 4. Discussion

Understanding the underlying pathological pathways and developing new therapies for a complex disease, such as AMD, remain challenging. So far, in vivo studies have been considered the best option to recapitulate in vivo response, providing new insights into the mechanisms involved in AMD and platforms for testing preclinical therapeutics [6]. Due to practical (financial, time, and facility resources) and especially ethical (the “Three Rs” principle when using laboratory animals) reasons, animal models should be replaced by alternative approaches, such as a 3D in vitro model. To date, the lack of suitable in vitro retinal models limits the replacement of animal experiments in AMD studies. Our research focuses on developing a 3D in vitro model, including both retinal and BrM models, to investigate the physiopathology of AMD and to test new treatments. To this end, we propose a multi-step approach. Here we presented the first two steps: *i*. the biomechanical characterization of the native retinal tissue, and *ii*. the development of a BrM model as a substrate for the first layer of retina composed of RPE cells.

Determining the biomechanical behavior of a tissue is essential to building an accurate in vitro model of that specific tissue. The mechanical properties of the retina are yet to be determined as only a few studies have examined them, probably due to the extreme structural fragility of retinal tissue [20,22]. In line with previous studies, we tested porcine retinas, which are structurally similar to human [23,24]. Porcine eyes are largely available, thus simpler to collect, and are often used as a precursor to experiments using human cadaveric specimens. Due to the similar anatomy, we hypothesized that the biomechanics of porcine retina could be translated to human. However, to the best of our knowledge, the mechanical behaviors of porcine and human retinas have not been compared yet. Considering the tests (*n* = 16) that we performed on porcine retinas, we can hypothesize that retinal biomechanical behavior is characterized by a short linear region of elastic deformation and a relatively wide non-linear region of plastic deformation, presenting several peaks before rupture. We propose that the peaks in the plastic region may reflect micro-ruptures in the tissue. These findings are consistent with the previous results of other authors [20,22]. Wollensack suggested that the remarkable wide range of the plastic phase with irreversible deformation but no tearing might represent a protective factor against tear formation [20]. Thanks to these outcomes, we will develop an in vitro retinal model that better replicates the native tissue. In fact, knowing retina biomechanics will help us to select a material with appropriate mechanical properties to develop a suitable bioink for the 3D Bioprinting of retinal cellular layers.

Accumulating evidence suggests that a BrM model is necessary to support the formation of a functional monolayer of RPE and to build a suitable in vitro model of AMD, since the BrM is also affected by thickening in the AMD [25]. Many artificial substrates have served as substitutes of BrM; however, these substrates still have disadvantages due to their incomparable ultrastructure and reduced ability to support RPE cells after long-term culture [10]. As such, an ideal substrate for a BrM model has yet to be found. The perfect BrM-mimetic scaffold should be as thin as the native BrM, have a porous permeable ultrastructure to allow the movement of nutrients and metabolic waste, be biocompatible, and exhibit a non-linear stress/strain behavior with a Young’s modulus of 6–14 MPa consistently with the native tissue, as established by computational models [26]. In fact, human BrM has proven to be difficult to isolate and manipulate for mechanical testing.

In this study, BMSF and PCL were selected to prepare the BrM in vitro model through electrospinning for two reasons. First, these two materials are biocompatible; BMSF is a natural material and PCL is approved by the Food and Drug Administration (FDA). Second, according to previous studies, both the PCL and SF scaffolds appeared to be advantageous in RPE culture experiments [18]. The resulting BMSF/PCL scaffolds showed structural and mechanical similarity to human BM.

The obtained electrospun scaffolds demonstrated a “nature-like” random nanofibrillar architecture with interconnected pores, as determined by SEM micrographs, which would allow the permeability of oxygen and the exchange of nutrients and metabolic waste, thereby mimicking the topographic features of the native BrM [16]. The permeability of the scaffolds was also confirmed by the permeability test carried out in this study, and the hydraulic conductivity was shown to be similar to that of native Bruch’s membrane [21]. According to Warnke et al., the native inner collagenous layer of a human BrM has a random fibrillary network, a packing density of 48%, and an average fiber diameter of 60 nm [16]. The average fiber diameter of our structures was in the order of nanometers, as reported by previous studies; however, further fine-tuning might be required in order to better match those of human BrM and to increase the permeability for a BrM in vitro model of AMD [27]. As far as for the thickness of the resultant scaffolds is concerned, as indicated by previous published data, importantly, our electrospun BrM scaffolds can be consistently produced in a range of desired thicknesses, comparable to the natural BrM in both physiological and pathological conditions, simply by regulating the electrospinning parameters, such as the process time [27]. The mechanical properties of the artificial BrM-mimetic substrates have been rarely investigated [16,17,19]. Zhang et al. reported that SF:PLCL (1:1) scaffolds had an elastic modulus of 105 ± 17.4 MPa, which is greater than that of our BMSF/PCL scaffolds [18]. Our results showed that the elastic modulus of the BMSF/PCL scaffolds developed in this study is closer to that of native BrM [26]. Finally, our electrospun scaffolds proved to be biocompatible. ARPE-19 cells were found to adhere well and spread on the scaffold surfaces. Further long-term research is needed to evaluate the formation of a functional epithelium.

Moreover, nanoindentation techniques will be explored to characterize the local stiffness sensed by the cells in both the native retina and the proposed scaffolds.

## 5. Conclusions

In this study, we have first characterized the biomechanical behavior of the retina to build a “Three Rs”-compliant in vitro retinal model with the same mechanical properties as the native tissue. Our findings have shown that the native retina presents a short elastic phase and a remarkably broad plastic phase with irreversible deformation. Secondly, we highlighted the importance of the inclusion of a BrM model in the in vitro retinal model to obtain a functional RPE monolayer and to better replicate AMD pathology. To this end, novel BrM-mimetic substrates were prepared from BMSF and PCL with a weight ratio of 5:95 via electrospinning. We have shown that the resultant substrates displayed structural and mechanical similarity to physiological BrM. Moreover, the substrate thickness can be easily adjusted to replicate pathological conditions. We demonstrated that the ARPE-19 cells seeded on our BMSF/PCL scaffolds attached, adhered, and spread. All these results are encouraging, and indicate that the produced electrospun scaffolds can be used as a BrM model and, thus, as a substrate for RPE cell culture.

## References

[1] Colijn J.M., Buitendijk G.H.S., Prokofyeva E., Alves D., Cachulo M.L., Khawaja A.P., Cougnard-Gregoire A., Merle B.M.J., Korb C., Erke M.G. (2017). Prevalence of age-related macular degeneration in Europe. Ophthalmology.

[2] Apte R.S. (2016). Targeting tissue lipids in age-related macular degeneration. EBioMedicine.

[3] Hunt N.C., Hallam D., Chichagova V., Steel D.H., Lako M. (2018). The application of biomaterials to tissue engineering neural retina and retinal pigment epithelium. Adv. Healthc. Mater..

[4] Guymer R., Luu C. (2014). Foreword: Age-Related macular degeneration. Curr. Issues Age Relat. Macular Degener..

[5] Sobrin L., Seddon J.M. (2014). Nature and nurture- genes and environment- predict onset and progression of macular degeneration. Prog. Retin. Eye Res..

[6] Pennesi M.E., Neuringer M., Courtney R.J. (2012). Animals model of age related macular degeneration. Mol. Asp. Med..

[7] Abokyi S., To C.H., Lam T.T., Tse D.Y. (2020). Central role of oxidative stress in age-related macular degeneration: Evidence from a review of the molecular mechanisms and animal models. Oxid. Med. Cell. Longev..

[8] Fenwick N., Griffin G., Gauthier C. (2009). Animal welfare. The welfare of animals used in science: How the “Three Rs” ethic guides improvements. CVJ.

[9] Publications Office of the European Union. https://op.europa.eu/en/publication-detail/-/publication/04a890d4-47ff-11ea-b81b-01aa75ed71a1.

[10] Tan Y.S.E., Shi P.J., Choo C.J., Laude A., Yeong W.Y. (2018). Tissue engineering of retina and Bruch’s membrane: A review of cells, materials and processes. Br. J. Ophthalmol..

[11] Hynes S.R., Lavik E.B. (2010). A tissue-engineered approach towards retinal repair: Scaffolds for cell transplantation to the subretinal space. Graefe’s Arch. Clin. Exp. Ophthalmol..

[12] Srivastava G.K., Martín L., Singh A.K., Fernandez-Bueno I., Gayoso M.J., Garcia-Gutierrez M.T., Girotti A., Alonso M., Rodríguez-Cabello J.C., Pastor J.C. (2011). Elastin-Like recombinamers as substrates for retinal pigment epithelial cell growth. J. Biomed. Mater. Res..

[13] Santos E., Hernández R.M., Pedraz J.L., Orive G. (2012). Novel advances in the design of three-dimensional bio-scaffolds to control cell fate: Translation from 2D to 3D. Trends Biotechnol..

[14] Lu J.T., Lee C.J., Bent S.F., Fishman H.A., Sabelman E.E. (2007). Thin collagen film scaffolds for retinal epithelial cell culture. Biomaterials.

[15] Asnaghi M.A., Candiani G., Farè S., Fiore G.B., Petrini P., Raimondi M.T., Soncini M., Mantero S. (2011). Trends in biomedical engineering: Focus on regenerative medicine. J. Appl. Biomater. Biomech..

[16] Warnke P.H., Alamein M., Skabo S., Stephens S., Bourke R., Heiner P., Liu Q. (2013). Primordium of an artificial Bruch’s membrane made of nanofibers for engineering of retinal pigment epithelium cell monolayers. Acta Biomater..

[17] Xiang P., Wu K.C., Zhu Y., Xiang L., Li C., Chen D.L., Chen F., Xu G., Wang A., Li M. (2014). A novel Bruch’s membrane-mimetic electrospun substrate scaffold for human retinal pigment epithelium cells. Biomaterials.

[18] Zhang D., Ni N., Chen J., Yao Q., Shen B., Zhang Y., Zhu M., Wang Z., Ruan J., Wang J. (2015). Electrospun SF/PLCL nanofibrous membrane: A potential scaffold for retinal progenitor cell proliferation and differentiation. Sci. Rep..

[19] Popelka Š., Studenovská H., Abelová L., Ardan T., Studený P., Straňák Z., Klíma J., Dvořánková B., Kotek J., Hodan J. (2015). A frame-supported ultrathin electrospun polymer membrane for transplantation of retinal pigment epithelial cells. Biomed. Mater..

[20] Wollensak G., Spoerl E. (2004). Biomechanical characteristics of retina. Retina J. Ret. Vit. Dis..

[21] Moore D.J., Hussain A.A., Marshall J. (1995). Age-Related variation in hydraulic conductivity of Bruch’s membrane. Investig. Ophthalmol. Vis. Sci..

[22] Chen K., Weiland J.D. (2010). Anisotropic and inhomogeneous mechanical characteristics of the retina. J. Biomech..

[23] Beauchemin M.L. (1974). The fine structure of the pig’s retina. Albrecht Graefes Arch. Klin. Ophthalmol..

[24] Chandler M.J., Smith P.J., Samuelson D.A., MacKay E.O. (1999). Photoreceptor density of the domestic pig retina. Vet. Ophthalmol..

[25] Curcio C.A., Johnson M., Schachat A.P., Wilkinson C.P., Hinton D.R., Sadda S., Wiedemann P. (2013). Structure, function, and pathology of Bruch’s membrane. Retina.

[26] Chan W.H., Hussain A.A., Marshall J. (2007). Youngs modulus of Bruchs membrane: Implications for AMD. Investig. Ophthalmol. Vis. Sci..

[27] Fridrikh S.V., Yu J.H., Brenner M.P., Rutledge G.C. (2003). Controlling the fiber diameter during electrospinning. Phys. Rev. Lett..

